# Bibliometric analysis of publication trends and topics of influenza‐related encephalopathy from 2000 to 2022

**DOI:** 10.1002/iid3.1013

**Published:** 2023-09-20

**Authors:** Zhengyu Zhang, Juntao Tan, Ying Li, Xiumei Zhou, Jianhua Niu, Jun Chen, Hongfeng Sheng, Xiaoxin Wu, Yuan Yuan

**Affiliations:** ^1^ Medical Records Department, The First Affiliated Hospital Zhejiang University School of Medicine Hangzhou China; ^2^ Operation Management Office Affiliated Banan Hospital of Chongqing Medical University Chongqing China; ^3^ Department of Medical Administration, The First Affiliated Hospital Zhejiang University School of Medicine Hangzhou China; ^4^ Department of Infectious Diseases People's Hospital of Pujiang County Zhejiang China; ^5^ PuJiang branch of the First Affiliated Hospital Zhejiang University School of Medicine Jinhua China; ^6^ Intensive Care Department, The First Affiliated Hospital Zhejiang University School of Medicine Hangzhou China; ^7^ Lung Transplant Department, The First Affiliated Hospital Zhejiang University School of Medicine Hangzhou China; ^8^ Department of Orthopedics Tongde Hospital of Zhejiang Province Hangzhou China; ^9^ State Key Laboratory for Diagnosis and Treatment of Infectious Diseases The First Affiliated Hospital, Zhejiang University School of Medicine, National Clinical Research Centre for Infectious Diseases Hangzhou Zhejiang China; ^10^ Medical Records Department Women and Children's Hospital of Chongqing Medical University Chongqing China

**Keywords:** bibliometric, CiteSpace, data visualization, influenza‐related encephalopathy, VOSviewer

## Abstract

**Background:**

Influenza‐related encephalopathy is a rapidly progressive encephalopathy that usually presents during the early phase of influenza infection and primarily manifests as central nervous system dysfunction. This study aimed to analyze the current research status and hotspots of influenza‐related encephalopathy since 2000 through bibliometrics analysis.

**Methods:**

The Web of Science Core Collection (WOSCC) was used to extract global papers on influenza‐related encephalopathy from 2000 to 2022. Meanwhile, the VOSviewer and CiteSpace software were used for data processing and result visualization.

**Results:**

A total of 561 published articles were included in the study. Japan was the country that published the most articles, with 205 articles, followed by the United States and China. Okayama University and Tokyo Medical University published the most articles, followed by Nagoya University, Tokyo University, and Juntendo University. Based on the analysis of keywords, four clusters with different research directions were identified: “Prevalence of H1N1 virus and the occurrence of neurological complications in different age groups,” “mechanism of brain and central nervous system response after influenza virus infection,” “various acute encephalopathy” and “diagnostic indicators of influenza‐related encephalopathy.”

**Conclusions:**

The research progress, hotspots, and frontiers on influenza‐related encephalopathy after 2000 were described through the visualization of bibliometrics. The findings will lay the groundwork for future studies and provide a reference for influenza‐related encephalopathy. Research on influenza‐related encephalopathy is basically at a stable stage, and the number of research results is related to outbreaks of the influenza virus.

## Graphical abstract

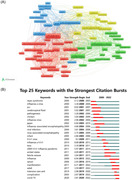

## 1 INTRODUCTION

Encephalopathy is an umbrella term that refers to temporary or permanent disturbances of brain functions. Indeed, a broad range of diseases can cause encephalopathy, including viral infection.^1^ Influenza is a highly infectious disease caused by a single‐stranded RNA virus and is a leading cause of disease and death worldwide. It is estimated that there are 1 billion cases and 290,000–650,000 influenza‐related respiratory deaths occurring every year.^2^ There are three common types of influenza viruses; influenza A and B viruses lead to flu syndrome, including acute upper respiratory illness, cough, fever, headache, and myalgia. Influenza C infection is usually asymptomatic.^3^ There are two kinds of virus glycoprotein spikes (haemagglutinin and neuraminidase) on the surface of influenza A or B virus particles, and alterations of neuraminidase with neurovirulence have been reported.^4^ Although most patients recover completely from influenza, there are short‐ and long‐term consequences for the central nervous system (CNS). The most commonly encountered extra‐respiratory complication is encephalopathy, which usually occurs 1 week following the first symptoms of influenza.^5^ Influenza‐related encephalopathy is a rapidly progressive encephalopathy that usually presents in the early phase of influenza infection and primarily induces central nervous system dysfunction.^6^

Since 1918, various neurological and cognitive effects have been associated with influenza infection.^2^ At least 12 major influenza pandemics have been recorded in the literature in the 20th and 21st centuries. Encephalitis, ataxia, and seizures were the most prevalent encephalopathy‐related disorders.^7^ In 1985, Japanese scholars first paid attention to the increase in the incidence rate of acute encephalopathy in children during the influenza epidemic, which was approximated to be 100–500 cases per year.^8^, ^9^ The global outbreak of influenza A H1N1 in 2009 has been proven to be more neurotoxic; more than 50% of H1N1 infected patients experienced neurological symptoms, while 9% of patients suffered from neurological complications.^10^, ^11^ Among them, children (especially those under 5‐year‐old) are more prone to neurological complications and have a higher mortality.^12^, ^13^

At present, sporadic outbreaks of seasonal influenza occur in numerous countries and pose a continuous threat to public health.^14^ Although influenza‐related encephalopathy is a relatively uncommon severe complication following influenza, influenza has brought about a considerable burden on the central nervous system of afflicted infants, children, and adolescents, which is highly concerning.^6^ Therefore, this study aimed to conduct an analysis of influenza‐related encephalopathy literature through bibliometric analysis to further elucidate the research status and development trend of influenza‐related encephalopathy‐based research.^15^

Literature on influenza‐related encephalopathy was reviewed based on the Web of Science Core Collection (WoSCC) of the Institute for Scientific Information (ISI). The analytical software VOSviewer and CiteSpace were used to: (Ⅰ) Perform bibliometric analyses of influenza‐related encephalopathy studies published since 2000; (Ⅱ) Draw visual knowledge maps; (Ⅲ) Display the research status of influenza‐related encephalopathy; (Ⅳ) Reveal research hotspots and development trends; (Ⅴ) Provide a reference for future relevant research.

## 2 MATERIALS AND METHODS

### 2.1 Data collection

**Figure 1:**
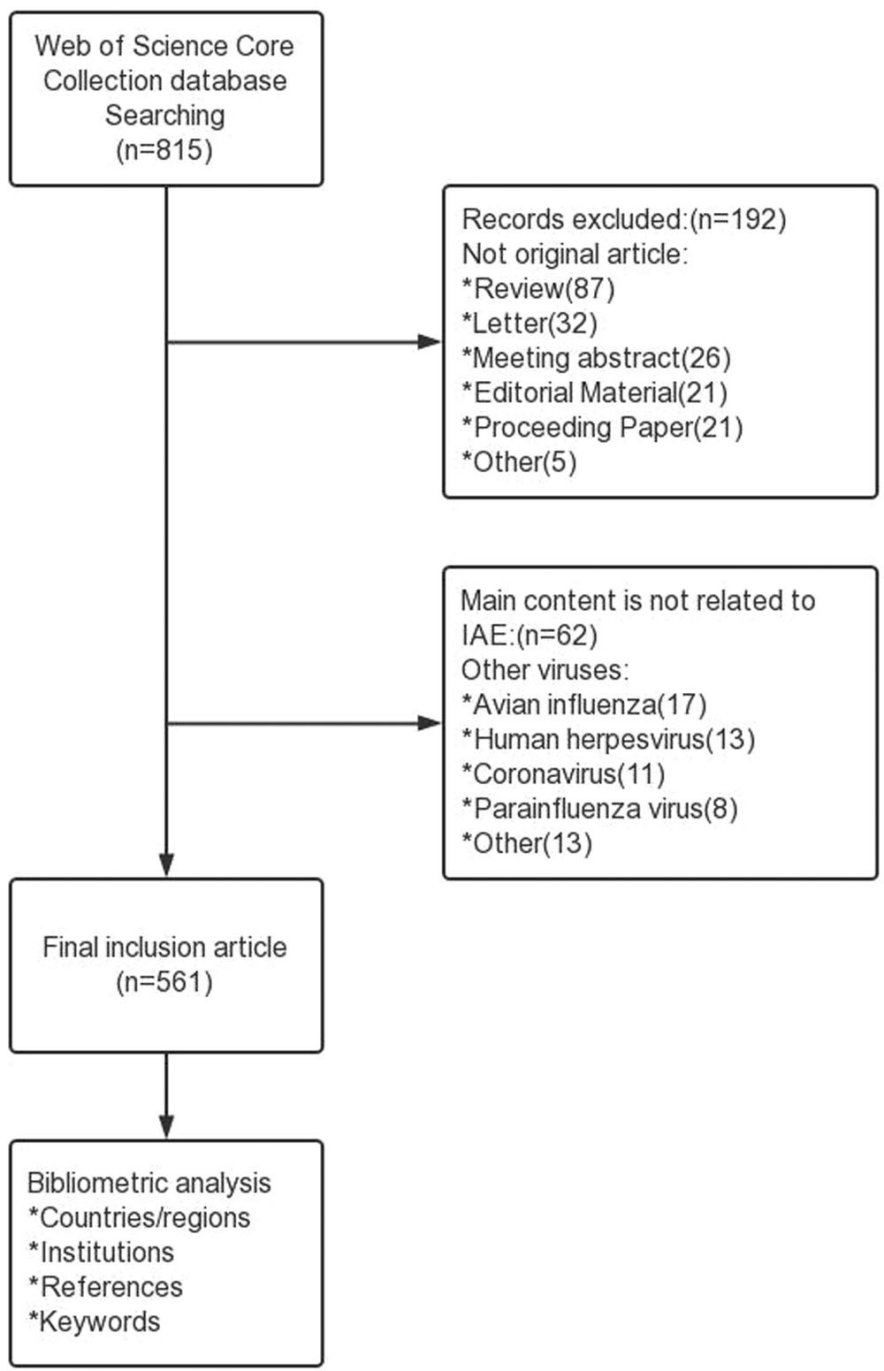
Flow chart of literature inclusion and exclusion.

The following search terms were used to retrieve literature from the Web of Science Core Citation (WOSCC) database: Topic = (“influenza” and “encephalopathy”). The search period was from January 1, 2000, to December 31, 2022. Only original research articles were included in the analysis, and there was no restriction on language. To ensure that the data were not being updated, two researchers completed the retrieval and screening of literature on the same day. The flow chart is illustrated in Figure 1. The data of this study was acquired from an open‐source database with no patients involved; thus, no ethical approval was required.

### 2.2 Analysis methods and visualization

VOSviewer software (version 1.6.19) and CiteSpace software (version 6.1.6R, 64bit) were used for data processing and result visualization. VOSviewer (a scientific knowledge graph application software) describes the structure, progression, coordination, and other aspects of the knowledge field by constructing and visually analyzing key terms extracted from scientific documents.^16^ In this study, VOSviewer was used to draw the visual knowledge distribution map: (1) co‐authorship of countries, authors, and research institutions; (2) co‐occurrence of keywords. Citespace is an interactive visualization tool developed based on Java and combines bibliometrics, data mining algorithms, and information visualization methods. It uses clustering functions to visually display the subject foundation and research hotspots.^17^ In this study, Citespace was used to extract references and keywords with periodic bursts.

## 3 RESULTS

**Figure 2:**
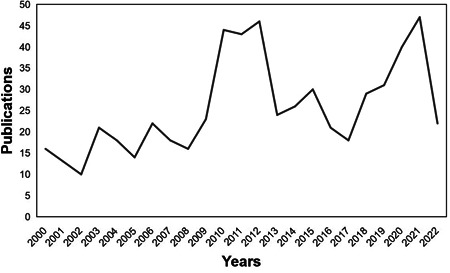
Trends in the growth of publications from 2010 to 2022.

According to the WOSCC database search results, a total of 561 published articles were included in our analysis. The number of publications in a period reflects the development trend of the research.^18^ The annual publications on IAE from 2000 to 2022 are listed in Figure 2. The number of studies was generally similar across all time periods, except for a temporary increase in the number of publications between 2010–2012 and 2020–2021.

### 3.1 Geographic trends

**Figure 3:**
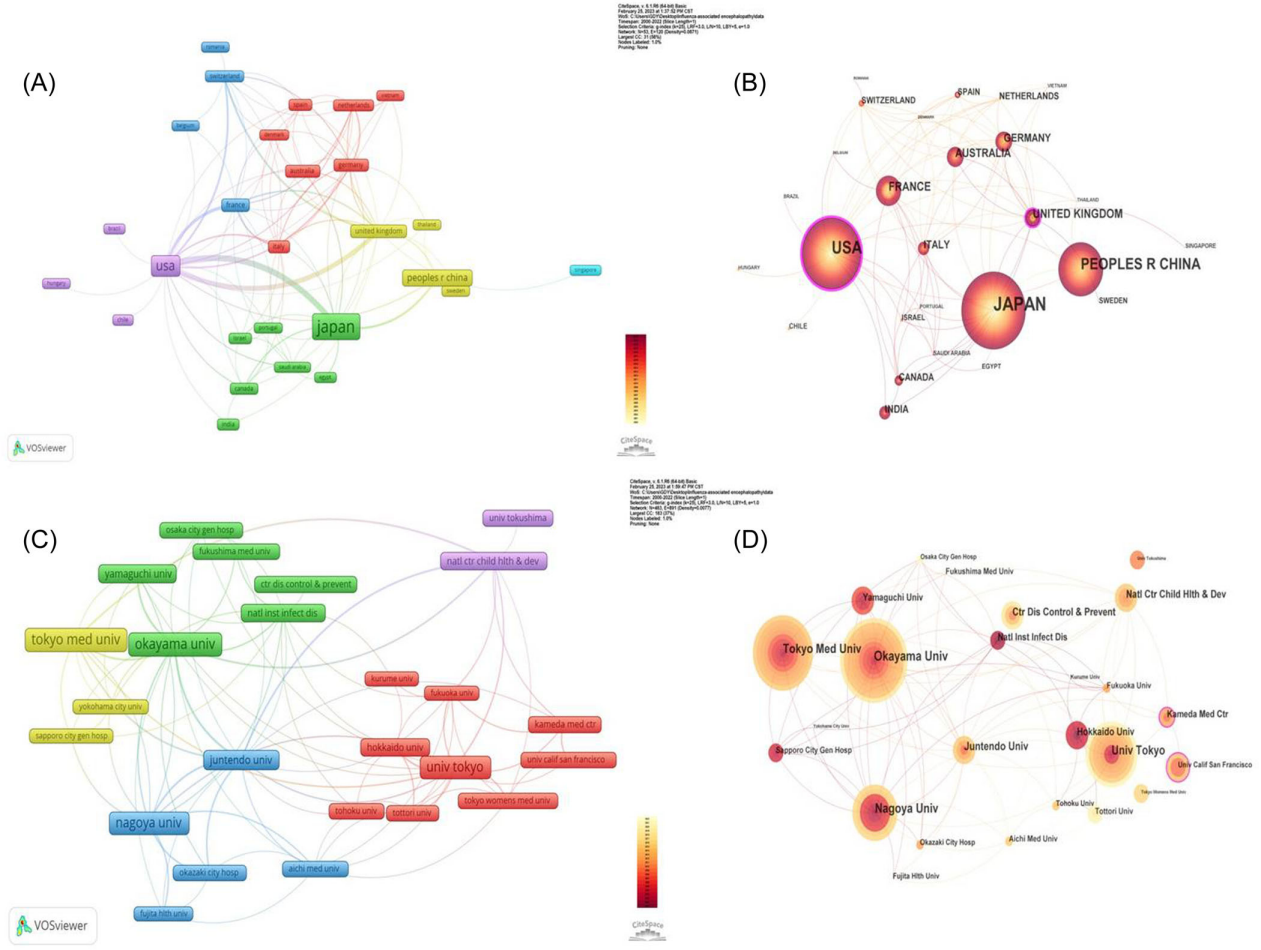
Cooperation visualization map of countries and institutions in the studies of IAE. (A) Cooperation clustering map of countries. (B) Centrality map of countries. (C)Cooperation clustering map of institutions. (D) Centrality map of institutions.

A total of 796 institutions from 54 countries have published articles on IAE. The top 10 countries and institutions that published articles related to IAE are presented in Tables 1 and 2. *Japan* was the country with the highest number of published articles, with 205 articles, followed by *the United States* (*n* = 125), *People R China* (*n* = 66), *France* (*n* = 26), and the *United Kingdom* (*n* = 24). The cooperation networks of countries are depicted in Figure 3A,B. Meanwhile, the United States had the highest centrality (0.24), displaying that it cooperated most with other countries. In Japan, *Okayama University* and *Tokyo Medical University* published the highest number of articles (*n* = 26), followed by *Nagoya University* (*n* = 24), *University of Tokyo* (*n* = 23), and *Juntendo University* (*n* = 15). Moreover, the top 10 institutions were all located in Japan, suggesting that Japan has several outstanding research groups in this field. The cooperation network of institutions with more than five articles is shown in Figure 3C,D. During the period from 2000 to 2022, most parts of the research cooperation on IAE were carried out by institutions in Japan. The institutions with the most international cooperation in IAE research were *Kameda Medical Center* (Japan, articles = 8, centrality = 0.14), and *University of California, San Francisco* (United States, articles = 5, centrality = 0.13).

**Table 1:** The top 10 countries in number of publications concerning IAE.

Rank	Country	Number of documents	Total citations	Centrality
1st	Japan	205	6145	0.05
2nd	The United States	125	5391	0.24
3rd	People's R China	66	1045	0.01
4th	France	26	793	0.03
5th	United Kingdom	24	1464	0.19
6th	Australia	21	633	0.00
7th	Italy	18	801	0.02
8th	Germany	17	725	0.01
9th	Turkey	17	227	0.00
10th	India	15	157	0.00

**Table 2:** The top 10 institutions in number of publications concerning IAE.

Rank	Institution	Country	Number of documents	Total citations	Centrality
1st	Okayama University	Japan	26	576	0.02
2nd	Tokyo Medical University	Japan	26	359	0.01
3rd	Nagoya University	Japan	24	1177	0.01
4th	University of Tokyo	Japan	23	1211	0.04
5th	Juntendo University	Japan	15	561	0.06
6th	Yamaguchi University	Japan	14	1147	0.01
7th	National Center for Child Health and Development	Japan	12	464	0.02
8th	National Institute of Infectious Diseases	Japan	11	687	0.02
9th	Hokkaido University	Japan	10	485	0.00
10th	The University of Tokushima	Japan	10	366	0.00

### 3.2 Subtopic trends

Intellectual base and research fronts are two typical indicators of a research program in a research field.^19^ The intellectual base refers to all documents that have been cited by corresponding research fields, while the research frontier arises from the intellectual base of this field. Reference of all the academic works in a research field constitutes the foundation of that field. Through citation analysis, we can understand the intellectual base, discipline evolution process, and research frontiers of the field. In addition, research hotspots were further explored by extracting keywords.

**Figure 4:**
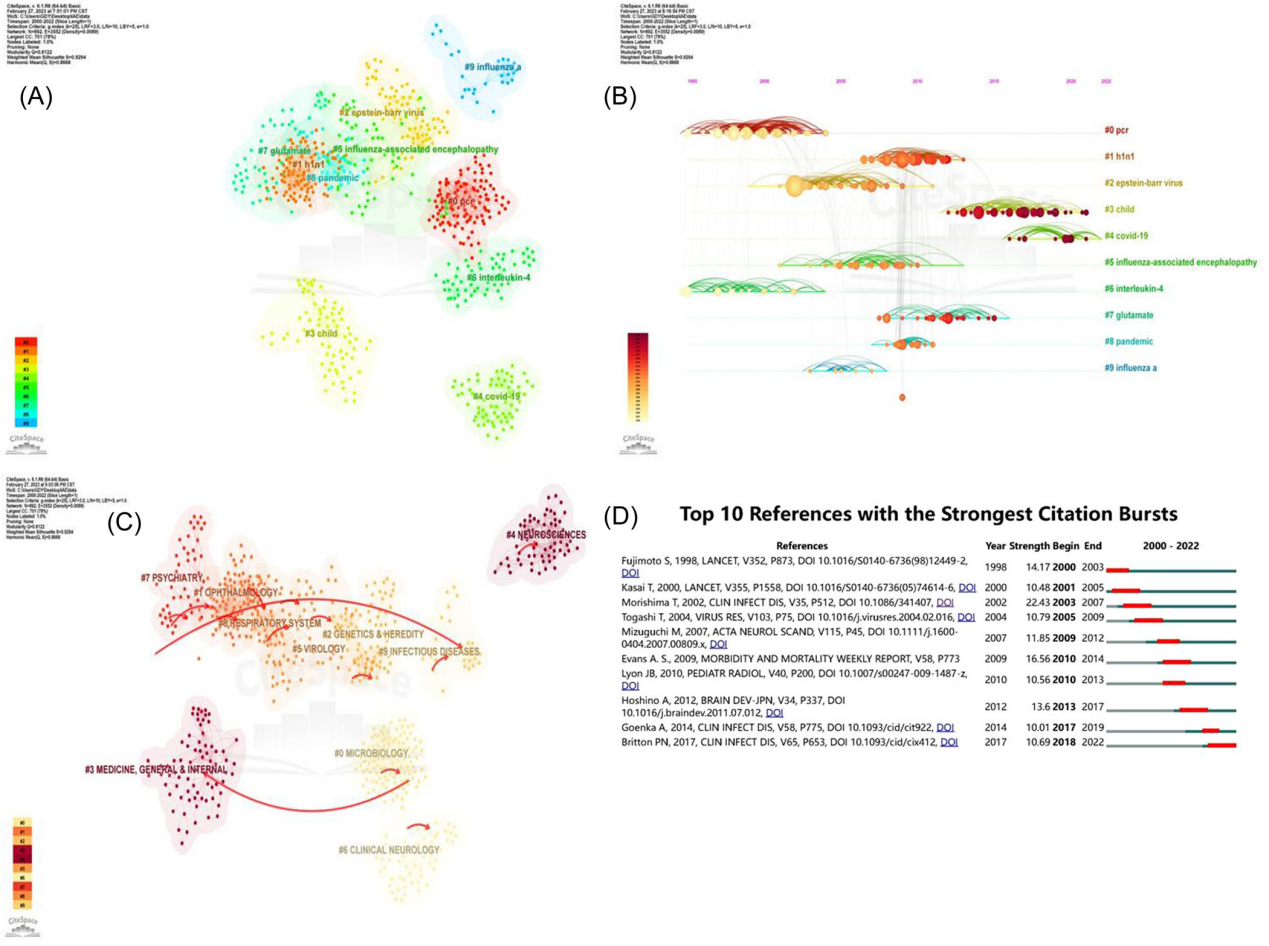
Analysis of references. (A) References co‐citation clustering network. (B) Timeline view of the co‐citation references. (C) Subject categories clustering network. (D) Top 10 references with the strongest citation bursts.

A total of 10,092 references were cited in 561 articles, and 10 major subtopics related to IAE were identified using the reference clustering function of Citespace. The co‐citation network analysis of references is depicted in Figure 4A. According to the analysis results, Modularity Q was 0.8122, and the weighted mean silhouette S was 0.9294, which are both considered very high. This implies that in terms of co‐citation clustering, the definition of specialty is clear, and the clustering results are reliable. Figure 4B outlines the timeline view of the cited citation references, which reflects the evolution pathway of each subtopic through time. The largest subtopic was # 0 PCR, and active subtopics in recent years were cluster # 3 child and # 4 covid‐19. # 5 influenza‐associated encephalopathy (IAE) first occurred in 2001, and this was followed by a burst of studies until 2013. The subject categories clustering network of references is shown in Figure 4C. The Modularity Q and weighted mean Silhouette S both demonstrated a great clustering effect and network homogeneity. The color from light to dark indicates the time from far to near, and the guiding line indicates the citation trend. According to the clustering results, # 3 MEDICINE, GENERAL & INTERNAL, and # 4 NEUROSCIENCES were the most prominent subjects of IAE‐related research in recent years. Figure 4D exhibits the top 10 references with the strongest citation bursts; the strength reflects the importance of this document to relevant fields.

### 3.3 Hotspots trends

**Figure 5:**
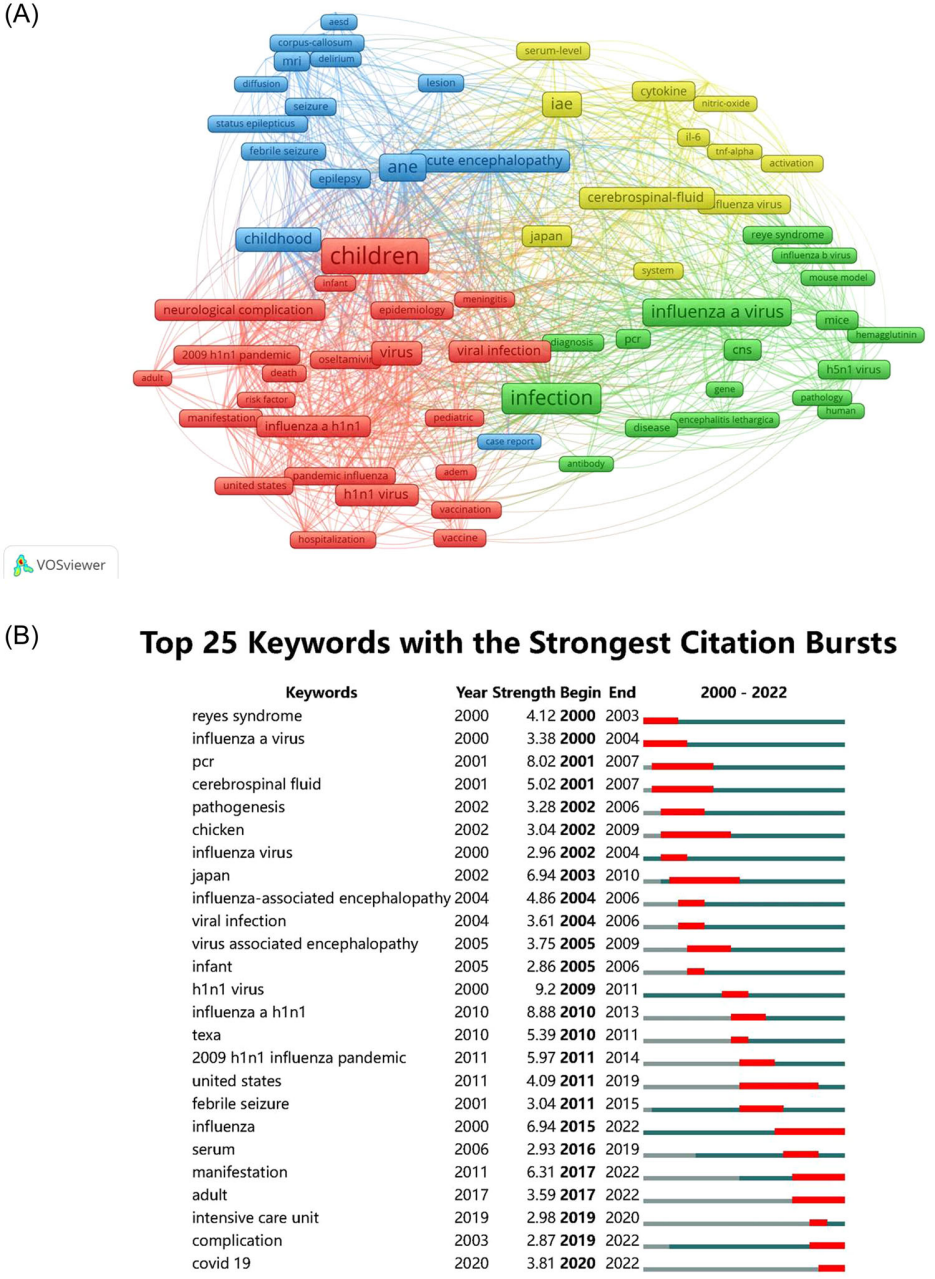
Analysis of keywords. (A) Map of keyword clustering in the studies of IAE. (B) Top 25 keywords with the strongest citation bursts. IAE, influenza‐associated encephalopathy.

The research patterns of keyword frequency were adopted to explore the hotspots trend in this study, and herein, the search words “influenza” and “encephalopathy” were excluded. The frequency of keywords such as child, infection, and influenza was high and used more than 100 times, while the frequency of other keywords such as acute necrotizing encephalopathy (ANE), IAE, virus, childhood, acute encephalopathy, h1n1 virus, and other related terms was reasonably high, with frequencies of over 50 times. The keyword clustering map developed by VOSviewer divided keywords with a total frequency of more than 10 times into 4 clusters (Figure 5A). Different color keywords represented different research directions. In this study, the largest cluster was the red cluster, which focused on the prevalence of the h1n1 virus, which causes neurological complications in different age groups, with the keywords “h1n1 virus,” “2009 h1n1 pandemic,” “children,” “adults,” and “neurological complications.” The second was the green cluster, which mainly explored the physiological and pathological reaction of the brain and central nervous system after influenza and chiefly included the keywords “infection,” “influenza a,” “brain,” and “central nervous system (CNS).” Contrastingly, the blue cluster mainly comprised various acute encephalopathies, represented by “epilepsy,” “seizure,” “acute necrotizing encephalopathy (ANE),” and “acute encephalopathy with biphasic seizures and late reduced diffusion (AESD).” Lastly, the keywords of the yellow cluster were related to the diagnostic indicators of IAE, such as “cerebrospinal‐fluid,” “cytokine,” and “serum‐level.” Keywords citation bursts are terms that occur abruptly in a short period of time or whose usage frequency dramatically increases. The top 25 keywords with the strongest citation bursts are shown in Figure 5B, and “h1n1 virus” had the strongest citation bursts (9.2). The citation bursts of keywords “influenza,” “manifestation,” “adult,” “complication,” and “covid‐19” ended in 2022. It is worthwhile pointing out that these keywords were the main contents of the current research.

## 4 DISCUSSION

IAE is a series of syndromes associated with central nervous system dysfunction in the acute phase of influenza, with high mortality and neurological sequelae, thereby posing a considerable threat to children hospitalized owing to influenza infection. However, complications involving the central nervous system (CNS) attributed to influenza have rarely attracted the attention of doctors. It was not until the early 1980s that Japanese scholars discerned that acute encephalopathy cases were clustered during the influenza epidemic and were a critical cause of influenza infection and death in Japanese children.^8^, ^20^ Until the end of the 20th century, acute encephalopathy related to influenza infection has been reported in a number of regions worldwide, including North America, Europe, and Australia.^21^, ^22^, ^23^

The results of this study signaled that the number of articles associated with influenza‐related encephalopathy was related to the epidemic status of influenza. Given that influenza‐related encephalopathy is a rare complication with an extremely low incidence rate (mean seasonal incidence rate: 2.8 cases/million for children; 0.19 cases/million for adults),^24^, ^25^ the number of clinical cases of influenza‐related encephalopathy outside the period of the noninfluenza pandemic is low, and thus the number of articles is relatively stable. The period between 2010 and 2012 was the peak of studies focusing on influenza‐related encephalopathy, which was closely linked to the global epidemic of the influenza A H1N1 strain first reported in the United States in 2009. It was the latest global epidemic of influenza, with more than 214 countries or regions globally reporting cases of influenza A H1N1, including over 18449 deaths (as of August 2010).^26^ We postulate that the increase in the number of articles from 2020 to 2021 may be correlated to the influenza A H1N1 epidemic in Asia, Europe, and other areas at the beginning of 2019^27^, ^28^, ^29^; another hypothesis is that this increase may also be associated with the fact that influenza‐related research had garnered more attention due to the global epidemic of COVID‐19 in 2020.^30^, ^31^

Japan started early in the research of influenza‐related encephalopathy with abundant and relatively mature research achievements. Indeed, it was the first country to publish reports on influenza‐related encephalopathy, with the largest number of documents and citations. Consequently, Japanese institutions occupy the leading position in this research field. The top 10 institutions with the highest number of documents were from Japan (Table 1), and most of the cooperation was conducted between Japanese institutions. This can be attributed to the improved epidemiological evaluation of influenza‐related acute encephalopathy in Japan and to the relatively high incidence of influenza‐related complications in Japan.^6^ It can be concluded from the three indicators (number of documents, total citations, and centrality) that Japan's ascendancy in influenza‐related encephalopathy is being gradually replaced by the United States.

Citation clustering demonstrated that the recent research subtopics of influenza‐related encephalopathy were “child” and “COVID‐19.” The reference trend of “Child” extended from 2011 to 2021. Epidemiological investigations and clinical case reports established that influenza‐related encephalopathy is most common in children and is the principal cause of influenza‐related childhood deaths.^8^, ^32^ The H1N1 strain in 2009 was more likely to lead to severe nervous system complications and long‐term sequelae than seasonal influenza.^10^ Surana et al.^33^ reported cases of children infected with the H1N1 virus during the H1N1 epidemic in 2009: 69% had characteristics of encephalopathy, 12% developed severe neurological sequelae, and 65% might have suffered from potential cognitive impairment. In comparison, Okumura and colleagues pointed out in the research report that 17% of the pediatric cases during the H1N1 epidemic in 2009 were accompanied by neurological sequelae, and 76% of the discharged children recovered and were discharged, but it is pivotal to track whether the use of antiviral drugs (oseltamivir) irreversibly increase the risk of consciousness disorders.^34^, ^35^ Therefore, long‐term follow‐up of children with influenza‐related encephalopathy may be one of the future research directions.

COVID‐19, the epidemic that broke out at the end of 2019, is the major public health emergency of the 21st century, with the fastest speed of transmission, the widest range of infection, and the greatest difficulty in prevention and control, resulting in more than 6 million deaths worldwide. It is an infectious respiratory disease that shares the same routes and means of transmission as the influenza virus and also possesses similarities in clinical characteristics and outcomes, laboratory and radiological manifestations.^36^, ^37^ It is worth noting that the overlap of COVID‐19 and the influenza virus during winter leads to co‐infection cases.^38^, ^39^ A systematic review conducted by Dadashi et al.^40^ exposed that among confirmed COVID‐19 patients, the prevalence of influenza infection was 0.8%, while the co‐infection rate of influenza virus in Asia and the United States was 4.5% and 0.4%, respectively. Although a low proportion of COVID‐19 patients suffered from influenza co‐infection, and the exact rate of co‐infection cannot be predicted, the superimposed effect of such co‐infection cannot be overlooked. Noteworthily, Alosaimi et al.^41^ described that influenza A H1N1 is the most common pathogen of co‐infection in COVID‐19 patients and the only pathogen positively correlated with mortality of COVID‐19 patients. Therefore, since 2020, the research on the comparison and identification of COVID‐19 and influenza in terms of transmission routes, diagnostic methods, clinical diagnosis, and disease prognosis, as well as surveillance and prevention measures of respiratory virus infections, has become research hotspots.^42^, ^43^, ^44^, ^45^

According to the evolution trend of the research subjects, neuroscience will be another research hotspot in the near future. This trend may be related to the increasing number of case reports of neurological characteristics following COVID‐19 infection. There is growing evidence that COVID‐19 provokes neurological and neuropsychiatric illnesses^46^; according to the results of Ellul et al., approximately 0.04% ~ 0.20% of COVID‐19 cases develop central nervous system complications while 0.05% ~ 0.16% suffer from peripheral nervous system complications.^47^ Although the majority of diagnoses regarding neurological damage due to COVID‐19 infection are still being made without laboratory validation, COVID‐19 infection might be a factor or co‐factor of long‐term neurological abnormalities to be revealed in later life.^48^

However, neither the literature included in this study nor the references cited by the literature indicate that COVID‐19 increases the incidence rate of influenza‐related encephalopathy. On the contrary, previous studies uncovered that the spread of influenza had been effectively controlled after countries had taken active measures to prevent COVID‐19 infections.^49^, ^50^, ^51^ For example, COVID‐19 outbreaks and related nonpharmaceutical interventions have reduced the incidence of influenza in Southern and Northern China and the United States by 79.2%, 79.4%, and 67.2%, respectively.^52^ The decrease in the influenza infection rate may be a potential cause for the decline in the number of publications on influenza‐related encephalopathy in 2022.

Herein, the keyword clustering network of the keyword analysis did not identify keywords related to the treatment strategy of influenza‐related encephalopathy, which may be related to a lack of specific treatments for influenza‐related encephalopathy.^2^ Typically, antiviral therapy is administered as soon as possible, but its role in enhancing neurological outcomes necessitates further investigation.^33^ The combination of high‐dose oseltamivir and glucocorticoids appears to be effective, but there are no international recommendations in terms of medication timing and dosage.^53^, ^54^, ^55^ Similarly, the study did not identify keywords about the pathogenesis of influenza‐related encephalopathy. At present, there are four hypotheses about the pathogenesis of influenza‐related encephalopathy: cytokine storm, viral invasion of the central nervous system, genetic predispositions, and activation of glial cells.^35^, ^56^ Hence, further international studies are warranted to elucidate its pathogenesis.

Nevertheless, seasonal influenza deserves attention, and many researchers are concerned about the alternate epidemics of influenza and COVID‐19. During the COVID‐19 period, there was a severe lack of screening and detection for influenza A.^57^, ^58^ Besides, adverse events of the COVID‐19 vaccine, such as neurotoxic side effects, led to a decrease in people's willingness to vaccinate against the influenza vaccine.^59^, ^60^, ^61^ Nonpharmaceutical interventions during the COVID‐19 epidemic may lower the body's immunity to the influenza A virus.^62^ These may be likely influencing factors that need to be paid more attention to in future research on influenza‐related encephalopathy.

## 5 CONCLUSION

Through the visualization of bibliometrics, the research progress, research hotspots, and research frontiers on influenza‐related encephalopathy after 2000 were described. The findings of this study further add to the available body of evidence and provide a reference for influenza‐related encephalopathy. Research on influenza‐related encephalopathy is currently at a stable stage, and the number of research results is related to outbreaks of influenza. For instance, the H1N1 influenza outbreak in 2009 has played a major role in the content of research on influenza‐related encephalopathy since 2010. Presently, Japan is the leader in the research on influenza‐related encephalopathy, but it is closely followed by the United States. The treatment strategy and pathogenesis of influenza‐related encephalopathy are critical issues that need to be addressed. COVID‐19 control measures have limited the spread of influenza, but the impact of the COVID‐19 pandemic on influenza‐related encephalopathy remains unknown. Research on influenza‐related encephalopathy is crucial, considering that its high‐risk population is children and its associated risk of sequelae such as brain injury.

## AUTHOR CONTRIBUTIONS

Zhengyu Zhang, Hongfeng Sheng, and Yuan Yuan designed the research. Yuan Yuan, Ying Li, Xiumei Zhou, Jianhua Niu, Jun Chen, and Zhengyu Zhang collected and organized data. Zhengyu Zhang and Yuan Yuan analyzed the data. Zhengyu Zhang, Juntao Tan, and Xiaoxin Wu drafted the manuscript. Yuan Yuan, Hongfeng Sheng, and Xiaoxin Wu contributed to the critical revision of the manuscript. All authors contributed to the manuscript and approved the submitted version.

## CONFLICT OF INTEREST STATEMENT

The authors declare no conflict of interest.

## Data Availability

The raw data supporting the conclusions of this article will be made available by the authors, without undue reservation.
